# Evaluation of *ATM* heterozygous mutations underlying individual differences in radiosensitivity using genome editing in human cultured cells

**DOI:** 10.1038/s41598-017-06393-8

**Published:** 2017-07-20

**Authors:** Ekaterina Royba, Tatsuo Miyamoto, Silvia Natsuko Akutsu, Kosuke Hosoba, Hiroshi Tauchi, Yoshiki Kudo, Satoshi Tashiro, Takashi Yamamoto, Shinya Matsuura

**Affiliations:** 1 Department of Genetics and Cell Biology, Research Institute for Radiation Biology and Medicine, Hiroshima University, Hiroshima, 734-8553 Japan; 2 Department of Biological Sciences, Faculty of Sciences, Ibaraki University, Mito, 310-8512 Japan; 3 Department of Obstetrics and Gynecology, Graduate School of Biomedical Sciences, Hiroshima University, Hiroshima, 734-8551 Japan; 4 Department of Cellular Biology, Research Institute for Radiation Biology and Medicine, Hiroshima University, Hiroshima, 734-8553 Japan; 5 Department of Mathematical and Life Sciences, Graduate School of Science, Hiroshima University, Higashi-Hiroshima, 739-8526 Japan

## Abstract

Ionizing radiation (IR) induces DNA double-strand breaks (DSBs), which are an initial step towards chromosomal aberrations and cell death. It has been suggested that there are individual differences in radiosensitivity within human populations, and that the variations in DNA repair genes might determine this heterogeneity. However, it is difficult to quantify the effect of genetic variants on the individual differences in radiosensitivity, since confounding factors such as smoking and the diverse genetic backgrounds within human populations affect radiosensitivity. To precisely quantify the effect of a genetic variation on radiosensitivity, we here used the CRISPR-ObLiGaRe (Obligate Ligation-Gated Recombination) method combined with the CRISPR/Cas9 system and a nonhomologous end joining (NHEJ)-mediated knock-in technique in human cultured cells with a uniform genetic background. We generated *ATM* heterozygous knock-out (*ATM* ^+/−^) cell clones as a carrier model of a radiation-hypersensitive autosomal-recessive disorder, ataxia-telangiectasia (A-T). Cytokinesis-blocked micronucleus assay and chromosome aberration assay showed that the radiosensitivity of *ATM* ^+/−^ cell clones was significantly higher than that of *ATM* ^+/+^ cells, suggesting that *ATM* gene variants are indeed involved in determining individual radiosensitivity. Importantly, the differences in radiosensitivity among the same genotype clones were small, unlike the individual differences in fibroblasts derived from A-T-affected family members.

## Introduction

To maintain genomic stability in human cells, the DNA damage response machinery recognises a variety of DNA lesions to orchestrate cellular fates such as DNA repair, cell cycle arrest and apoptosis^1^. DNA double-strand breaks (DSBs) induced by ionizing radiation (IR) lead to a huge loss of genetic information, which can cause carcinogenesis if they are left unrepaired. It has been shown that there are individual differences in the cellular capacity of DNA DSB repair within human populations^2, 3^, which we define cellular radiosensitivity in this study. The term “cellular radiosensitivity” is used to describe many different phenomena and is defined by the biological endpoints. Classically, cellular radiosensitivity is a measure of the cell killing to IR. Such cellular lethality to IR contributes to the occurrence of acute IR-induced tissue damages, while DNA DSB repair in early phase of DNA damage response influences the proneness to radiation-induced cancer.

The cellular capacity of DNA DSB repair can be assessed in many defferent assays. The cytokinesis-blocked micronucleus (CBMN) assay, which is an elaborate procedure to evaluate cellular radiosensitivity by counting micronuclei formed by unrepaired DSB-derived chromosomal fragments^4^, demonstrated the existence of mildly radiosensitive cases within a small population of healthy individuals and breast cancer patients^5^. The multi-colour fluorescent *in situ* hybridization (FISH) painting assay also revealed individual differences of IR-induced unstable chromosomal structural abnormalities including ring and dicentric chromosomes in healthy and cancer patient populations^6^. This heterogeneity might be attributable to variations in the DNA repair genes.

To clarify whether genetic variants in DNA repair genes are indeed associated with individual differences in radiosensitivity, it is informative to measure the radiosensitivity of primary cells with a genetic variant of interest, such as peripheral blood lymphocytes and skin fibroblasts. However, the radiosensitivity of human primary cells might be affected by confounding factors such as age, gender, smoking and the diverse genetic backgrounds within human populations. It is therefore necessary to generate a system for evaluating genetic factors underlying individual differences in radiosensitivity in a human cultured cell line with a uniform genetic background. Clustered regularly interspaced short palindromic repeats (CRISPR)/Cas9-mediated genome editing technology, which recognises the protospacer adjacent motif (PAM; 5′-NGG-3′) sequence and the region 20 bp upstream of it to introduce a DSB 3 bp upstream of the PAM sequence, enables a reverse genetics approach to be applied in human cultured cell lines with limited homologous recombination activity^7, 8^. Here, we demonstrate that the application of genome editing technology in human cultured cell lines could be useful to examine the biological effect of a genetic variant on radiosensitivity.

Ataxia-telangiectasia (A-T [MIM 607585]) is a rare autosomal-recessive disorder characterised by hyper-radiosensitivity, cancer predisposition, immunodeficiency and neurodegeneration^9^. A-T is caused by germline mutations in the *ataxia-telangiectasia mutated* (*ATM*) gene encoding ATM kinase, which is a DSB damage response master kinase member of the evolutionarily conserved phosphatidylinositol-3-kinase-related kinase (PIKK) family^10^. The cells of A-T patients exhibit severe genetic instability, high probability of malignant transformation and extreme sensitivity to radiation^10^. Previous epidemiological studies demonstrated that A-T heterozygous carriers showed a several-fold increased risk of breast and ovarian cancers in comparison with normal individuals^11^. Since A-T heterozygous carriers, who are clinically asymptomatic, exist at a rate of approximately 1% in human populations^9^, we assume that the heterozygous form of recessive mutations associated with hyper-radiosensitive genetic disorders such as A-T when in a homozygous state might be a genetic determinant of individual differences in healthy human populations. Previous studies indicated that most primary cells from A-T heterozygous carriers were more radiosensitive than those from normal individuals^12^, while it was also reported that the radiosensitivities of A-T heterozygous carriers and normal individuals were not segregated because of the genetic heterogeneity in some cases^13^. It is therefore important but difficult to quantify the precise effect of *ATM* heterozygous mutations on radiosensitivity in the primary cells.

To generate human *ATM* heterozygous and homozygous mutated-cultured cell clones with a uniform genetic background, we here used the “Obligate Ligation-Gated Recombination” (ObLiGaRe) approach, the original concept of which was reported by Maresca *et al*.^14^, which enabled the insertion of a drug-resistant gene cassette tagged with the genomic CRISPR/Cas9 recognition sequence into the specific *ATM* locus via NHEJ activity in the hTERT-RPE1 cell line from human normal retina pigmented cells. In this study, we demonstrated that semiautomated CBMN and chromosome aberration analyses in the CRISPR/ObLiGaRe-mediated model cells could quantify the effect of *ATM* heterozygous mutations on radiosensitivity.

## Results

### Semiautomatic CBMN assay in primary fibroblasts revealed individual differences in radiosensitivity in A-T-affected family members

**Figure 1:**
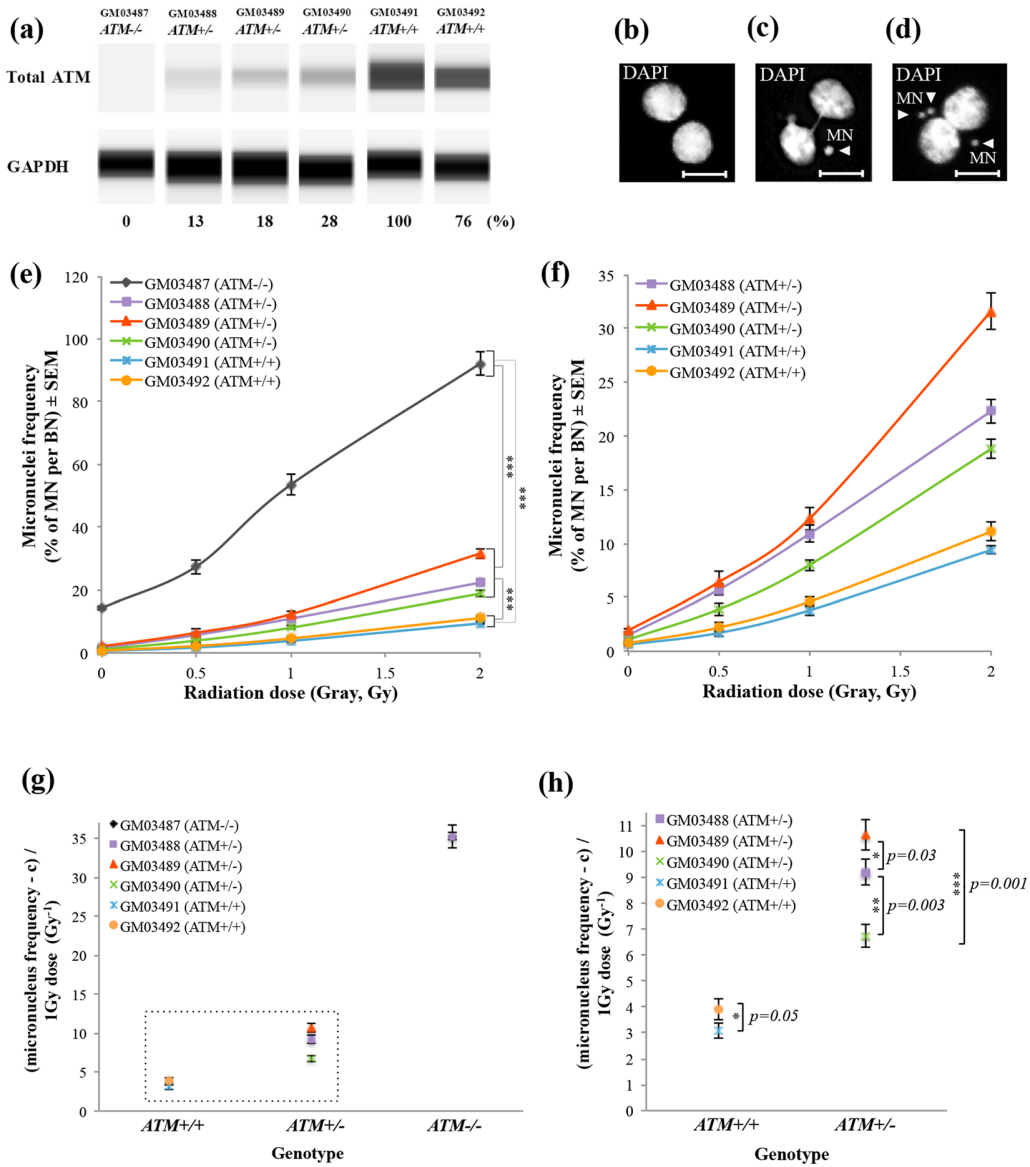
CBMN assays in primary fibroblasts detected the effect of different genetic backgrounds on radiosensitivity. (**a**) Western blotting analysis data showing the expression levels of ATM protein in primary fibroblasts derived from the six members of an A-T-affected family. The GAPDH antibody was used as a loading control. The intensity of ATM bands was normalized to that of GAPDH and is shown as a percentage regarding the score of GM03491, with the maximal ATM expression set as 100%. (**b**–**d**) Metafer MN Search images showing the cytokinesis-blocked fibroblasts stained with DAPI. BN cell without MN (b), BN cell with one MN (d); BN cell with three MN (e). Arrowheads indicate the MN. Scale bars: 10 μm. (e) Percentage of IR-induced MN formation in fibroblasts from all members of the A-T-affected family (mean ± SE; t-test; ***p < 0.001, **p < 0.01, *p < 0.05; n = 3; >1000 BN cells, >50 BN cells only in A-T patient fibroblasts). (**f**) The graph from (e) with magnification of the Y-axis including the percentage of IR-induced MN formation in fibroblasts derived from A-T heterozygous carriers and unaffected individuals. (**g**) Radiosensitivity in the six members of the A-T-affected family was quantified using the sum of α and β coefficients upon γ-ray irradiation at 1Gy (mean ± SE; t-test; n = 3; >1000 BN cells per experiment, >50 BN cells per experiment only in A-T patient fibroblasts). A-T patient cells showed a remarkable rasiosensitivity score. **(h)** The graph from (g) with magnification of the Y-axis including the radiosensitivity scores of A-T heterozygous carriers and normal individuals in the A-T-affected family. Significant inter-individual differences in radiosensitivity are denoted by asterisks (mean ± SE; t-test; ***p < 0.001, **p < 0.01, *p < 0.05; n = 3; >1000 BN cells per experiment). Cropped blots are displayed and the full-length blots are included in the Supplementary Information file.

We collected human skin fibroblasts from a family affected by A-T, consisting of one patient with compound heterozygous *ATM* null mutations (c.1141ins4, p.S381X; c.8266 A > T, K2756X), three heterozygous carriers and two normal individuals (Table S1). Fibroblasts from the patient had no ATM protein, while those from the heterozygous carriers showed significant reductions of ATM protein compared with the levels in the normal individuals (Fig. 1a, and Fig. S7a). Next, to verify that *ATM* heterozygous mutations are indeed involved in individual differences in radiosensitivity, we used the automatic Metafer system to detect micronuclei (MN) in the IR-treated binucleated (BN) cells, in which cytokinesis was blocked by cytochalasin-B (Fig. 1b–d). Automatically obtained images of MN were reanalysed visually (i.e., a semiautomatic approach) to remove pseudo-positive and/or negative MN and BN cells. To ensure more reliable results, more than 1000 images of BN cells were scored in each condition. However, we allowed fewer BN cells from the A-T patient to be counted due to the hyper-radiosensitive growth arrest and cell death. From three independent experimental trials (Fig. S1), we extracted the average of the ratio of MN to BN cells along with the standard error for each point and used these data to create representative dose-response calibration curves (Fig. 1e). As expected, the A-T patient cells exhibited more than 90% of micronucleus formation after 2 Gy of IR irradiation (Fig. 1e). Since the ratios of MN/BN cells of the A-T patient cells were much higher than the others, as shown in Fig. 1f, we magnified the axis of MN frequency for the curves of cells from the heterozygous carriers and normal individuals. Consistent with previous studies^12, 15^, IR-induced micronucleus formation in the A-T heterozygous carrier cells was significantly enhanced compared with that of normal individual cells (Fig. 1f). Importantly, individual differences of IR-induced micronucleus formation among the A-T heterozygous carriers and normal individuals were also detected, suggesting the significant heterogeneity of radiosensitivity even among those with the same *ATM* genotype.

To evaluate the radiosensitivity of cells from the family affected by A-T in a more quantitative manner, the dose-response curves of the ratio of MN/BN cells were analysed using a linear-quadratic model: MN frequency = c + αD + βD^2^. α, β, and c coefficients in each primary cell were extracted using chromosomal aberration calculation (Cabas) software^16^ (Table 1). This formula was then converted to (MN frequency − c)/D = α + βD. The mean data scores (D = 1 Gy) are plotted in Fig. 1g and h, indicating that the sum of α and β coefficients accurately reflected the radiosensitivity of cells to γ-ray irradiation at an intensity level of 1 Gy^17^. The quantitative scores of radiosensitivity for the cells from the family affected by A-T increased in a manner dependent on the copy number of *ATM* heterozygous mutations and they were specific to the individual, implying that the genetic background within members of such families might modify the radiosensitivity of each cell. It is therefore difficult to precisely quantify the effect of *ATM* heterozygous mutations on radiosensitivity using the primary cells.

**Table 1:** Individual radiosensitivity coefficients (α, β, c, and α + β) in primary fibroblasts from an A-T-affected family.

Cell line ID/Genotype	β ± SE × 10^−3^ (Gy^−2^)	α ± SE × 10^−3^ (Gy^−1^)	c ± SE × 10^−3^	Radiosensitivity score [α + β]
GM03487 (*ATM* ^*−/−*^)	5.255 ± 0.3	30.012 ± 1.23	14.033 ± 0.49	35.267 ± 1.53
GM03488 (*ATM* ^*+/−*^)	1.252 ± 0.01	7.943 ± 0.48	1.489 ± 0.34	9.195 ± 0.49
GM03489 (*ATM* ^*+/−*^)	4.157 ± 0.2	6.473 ± 0.39	1.909 ± 0.35	10.63 ± 0.59
GM03490 (*ATM* ^*+/−*^)	2.147 ± 0.29	4.587 ± 0.14	1.111 ± 0.38	6.734 ± 0.43
GM03491 (*ATM* ^*+/+*^)	1.377 ± 0.02	1.72 ± 0.29	0.552 ± 0.38	3.097 ± 0.31
GM03492 (*ATM* ^*+/+*^)	1.342 ± 0.31	2.538 ± 0.08	0.651 ± 0.39	3.88 ± 0.39

α, β, and c coefficients were extracted from dose-response calibration curves in Fig. 1(e) using Cabas software. α + β is equivalent to IR-induced micronucleus formation upon γ-ray irradiation at 1 Gy.

### Generation of model human cell lines with a uniform genetic background by the “CRISPR/ObLiGaRe” genome-editing approach

In the next part of the study, we attempted to generate model clonal cell lines with a uniform genetic background using genome editing technology in hTERT immortalized retinal pigmented epithelial cell lines (hTERT-RPE1). The CRISPR/ObLiGaRe method shown in Fig. S2a enables the introduction of a targeting vector tagged with the endogenous genomic CRISPR/Cas9 recognition site through NHEJ activity to a specific locus. We cointroduced a CRISPR/Cas9 driver plasmid expressing both short-guide RNA (sgRNA) to exon 11 in the *ATM* gene and Cas9, and a drug-resistant gene cassette (hsvTK-2A-Neo) vector tagged with the same *ATM* sgRNA sequence into hTERT-RPE1 cells. Then, we positively selected the transfected cells in neomycin-supplemented growth medium for 2 weeks to isolate the *ATM* homozygous and heterozygous cell clones efficiently.

For genotyping of the *ATM* gene in the neomycin-resistant clones, we applied two-step analysis. In the first step, we performed amplification of the target region of exon 11 in the *ATM* gene by PCR in order to determine whether or not a drug-resistant cassette is inserted into the *ATM* locus (Figs S2b and S7b). Since the direction of insertion of a drug-resistant gene cassette into the targeting locus could not be controlled in the CRISPR/ObLiGaRe method, we designed three different types of primer pair (Fig. S2a and b): primer pairs *A* (*ATM* exon 11 forward *Fp*/*Neo* ^*r*^-reverse primer *Np*) and *B* (*ATM* exon 11 reverse *Rp*/*Neo* ^*r*^-reverse primer *Np*) to detect the integration of a drug-resistant gene cassette into the *ATM* gene in forward and reverse orientations, respectively, and primer pair *C* (*ATM* exon 11 forward/*ATM* exon 11 reverse) to confirm lack of integration of the drug-resistant cassette. As shown in Fig. S2b, *ATM* ^−/−^ clone 2 and *ATM* ^*+/−*^ clone 2 had monoallelic integration of a drug-resistant gene cassette in forward and reverse orientations, respectively, suggesting that they were either *ATM* ^*+/−*^ or *ATM* ^−/−^ clones. In the second step, we performed direct Sanger sequencing of the primer pair *C*-mediated PCR product to check whether or not insertions or deletions had occurred in the second allele. Since the second allele in *ATM* ^−/−^ clone 2 carried a 1-bp insertion (*ATM* c.1653 ins T; V551X) at 3 bp upstream from the PAM sequence of the sgRNA targeting *ATM* exon 11 (Fig. S2c), this clone was indeed an *ATM* null mutant (Fig. S2d). In contrast, *ATM* ^*+/−*^ clone 2 had no alteration in the *ATM* gene in the second allele (Fig. S2c), indicating that this clone was indeed an *ATM* heterozygous mutant (Fig. S2d). In this study, we screened 211 clones to generate 7 clones of the *ATM* heterozygotes and 153 clones of the null mutants (Table 2, Table S2). During this screening, we also identified an *ATM* ^*+/+*^ clone with the random integration of a drug-resistant gene cassette outside the *ATM* locus (Fig. S2d). Thus, the CRISPR/ObLiGaRe method enabled the generation of model cell lines from the A-T patient and the heterozygous carriers with a uniform genetic background.

**Table 2:** Generation of *ATM*-edited hTERT-RPE1 cell clones using “CRISPR/ObLiGaRe” method.

Total number of screened clonal lines (211 clones)				
Clones with	Bi-allelic Neo^R^ insertion	Mono-allelic Neo^R^ insertion		Random Neo^R^ insertion
# clones	9/211 (4.3%)	151/211 (71.6%)		51/211 (24.2%)
		Allele 2 genotyping (by direct sequencing)		
		With Indel(s):144/211 (68.2%)	Wild type: 7/211 (3.3%)	
*ATM* genotype	*ATM* ^−/−^	*ATM* ^*−/−*^	*ATM* ^*+/−*^	*ATM* ^*+/+*^

A total of 211 neomycin-resistant clones were isolated. Two-step genotyping analysis revealed that 153 (72.5%) and 7 (3.3%) clones were *ATM* ^−/−^ and *ATM* ^*+/−*^, respectively.

### Semiautomatic CBMN assay in *ATM*-edited cell clones with a uniform genetic background quantifed the precise effect of *ATM* mutations on radiosensitivity

**Figure 2:**
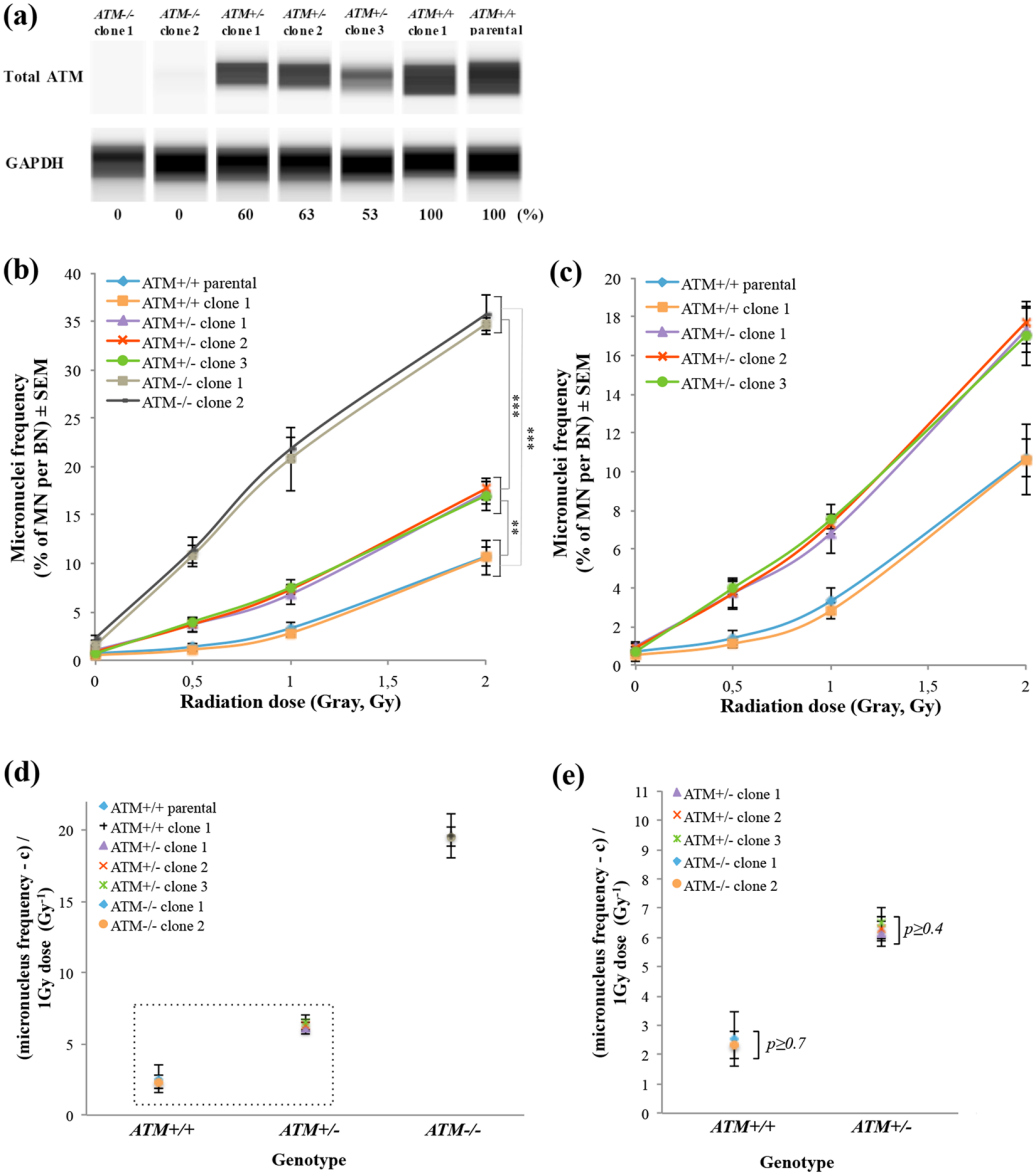
Screening approach for detecting the radiosensitivity of cells with a uniform genetic background. (**a**) Western blotting analysis data showing the expression levels of ATM protein in *ATM*-edited cell clones. The GAPDH antibody was used as a loading control. The intensity of ATM bands was normalized to that of GAPDH and is shown as a percentage relative to the score of *ATM* ^+/+^ cell clones set as 100%. (**b**) Percentage of IR-induced MN formation in *ATM*-edited cell clones (mean ± SE; t-test; ***p < 0.001, **p < 0.01, *p < 0.05; n = 3; >1000 BN cells, >50 BN cells only in *ATM* ^−/−^ cell clones). (**c**) The graph from (b) with magnification of the Y-axis including the percentage of IR-induced MN formation in *ATM* ^*+/−*^ and *ATM* ^+/+^ cell clones. (**d**) Radiosensitivity in *ATM*-edited cell clones was quantified using the sum of α and β coefficients upon γ-ray irradiation at 1 Gy (mean ± SE; t-test; n = 3; >1000 BN cells per experiment, >50 BN cells per experiment only in *ATM* ^−/−^ cell clones). *ATM* ^−/−^ cell clones exhibited a remarkable radiosensitivity score. (**e**) The graph from (d) with magnification of the Y-axis including the radiosensitivity scores of *ATM* ^*+/−*^ and *ATM* ^+/+^ cell clones. Significant clonal differences in radiosensitivity were not detected (mean ± SE; t-test; values of p > 0.05 were not significant; n = 3; >1000 BN cells per experiment).

Western blotting analysis revealed that *ATM* ^−/−^ clones had no signal of total ATM protein, while *ATM* ^*+/−*^ heterozygous clones showed reduced amounts of ATM protein compared with the parental hTERT-RPE1 cells and an *ATM* ^*+/+*^ clone (Fig. 2a, Fig. S7a). Thus, ATM protein expression levels in *ATM*-edited cell clones were consistent with the *ATM* genotypes. To quantify the effect of *ATM* mutations on radiosensitivity in a uniform genetic background, we applied the semiautomatic CBMN assay to a set of *ATM*-edited hTERT-RPE1 cell clones and investigated more than 1000 BN cells in three independent experimental trials (Fig. S3). *ATM* ^−/−^ cell clones showed extreme IR-induced micronucleus formation (Fig. 2b), while *ATM* ^*+/−*^ cell clones demonstrated a mildly radiosensitive phenotype in comparison with *ATM* ^*+/+*^ cell lines (Fig. 2c). Notably, the significant differences of IR-induced micronucleus formation among the clones with the same genotype were not detected, unlike the individual differences in fibroblasts from members of the family affected by A-T. To quantify the radiosensitivity of each cell clone from the CBMN assay, we used Cabas software to extract α, β, and c coefficients (Table 3). The radiosensitivity scores calculated by summing α and β in the model cell clones were dependent on the *ATM* genotype, while clonal variations of the scores in each genotype were not detected (Fig. 2d and e). Therefore, we calculated the ratio of the radiosensitivity scores of the *ATM* ^*+/−*^ clones to those of the *ATM* ^*+/+*^ clones to quantify the effect of *ATM* heterozygous mutations on radiosensitivity. Our results suggested that *ATM* heterozygous mutations contributed to an approximately 2.6-fold increase of radiosensitivity in normal human cells, and that they might be a genetic factor underlying the individual differences in radiosensitivity within human populations.

**Table 3:** Radiosensitivity coefficients (α, β, c, and α + β) in *ATM*-edited hTERT-RPE1 cell clones.

Cell line ID/Genotype	β ± SE × 10^−3^ (Gy^−2^)	α ± SE × 10^−3^ (Gy^−1^)	c ± SE × 10^−3^	Radiosensitivity score [α + β]
*ATM* ^*−/−*^ clone 1	−2.429 ± 0.06	21.979 ± 0.62	1.49 ± 0.39	19.55 ± 0.68
*ATM* ^*−/−*^ clone 2	−2.069 ± 0.43	21.68 ± 1.14	1.451 ± 0.78	19.611 ± 1.57
*ATM* ^*+/−*^ clone 1	1.951 ± 0.31	4.171 ± 0.11	0.979 ± 0.4	6.122 ± 0.42
*ATM* ^*+/−*^ clone 2	2.087 ± 0.27	4.196 ± 0.15	0.933 ± 0.39	6.283 ± 0.42
*ATM* ^*+/−*^ clone 3	1.398 ± 0.32	5.085 ± 0.2	0.733 ± 0.38	6.483 ± 0.52
*ATM* ^*+/+*^ clone 1	2.578 ± 0.09	−0.238 ± 0.39	0.53 ± 0.39	2.34 ± 0.48
*ATM* ^*+/+*^ parental	2.437 ± 0.4	0.099 ± 0.55	0.704 ± 0.37	2.536 ± 0.95

α, β, and c coefficients were extracted from dose-response calibration curves in Fig. 2(b) using Cabas software. Relative capacity to repair DNA after acute γ-irradiation was assessed at a dose of 1 Gy.

**Figure 3:**
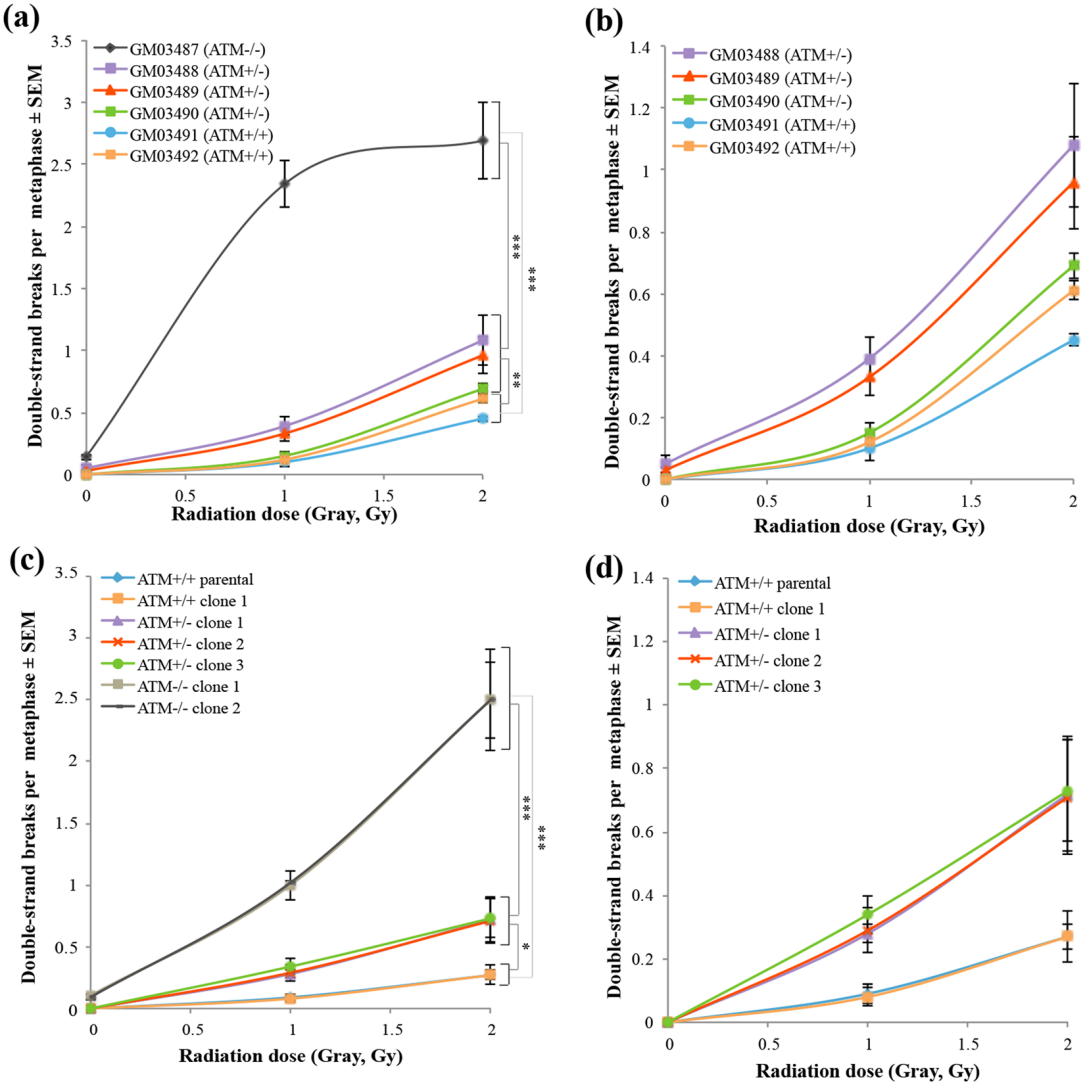
PNA-FISH analysis confirmed the effect of *ATM* heterozygous mutations on the cellular radiosensitivity in a different or uniformed genetic background. (**a**) Averaged unrepaired DSBs per metaphase in fibroblasts from all members of the A-T-affected family (mean ± SE; t-test; ***p < 0.001, **p < 0.01, *p < 0.05; values of p > 0.05 were not significant; n = 3; >100 metaphase cells per experiment). (**b**) The graph from (a) with magnification of the Y-axis including the percentage of averaged unrepaired DSBs per metaphase in fibroblasts from the A-T heterozygous carriers and normal individuals in the A-T-affected family. (**c**) Average unrepaired DSBs per metaphase in *ATM*-edited hTERT-RPE1 cell lines (mean ± SE; t-test; ***p < 0.001, **p < 0.01, *p < 0.05, values of p > 0.05 were not significant; n = 3; >100 metaphase cells per experiment). (**d**) The graph from (a) with magnification of the Y-axis including the percentage of averaged unrepaired DSBs per metaphase in *ATM* ^*+/−*^ and *ATM* ^+/+^ cell clones.

Chromosomal aberrations including dicentric/multicentric and ring chromosomes are also hallmarks of IR-induced unrepaired DNA lesions (Fig. S4). We thus used PNA-FISH probes to stain telomeres and centromeres quickly and clearly^18^, and then counted IR-induced chromosomal aberrations in cells derived from A-T-affected family members and *ATM*-edited hTERT-RPE1 clones. Consistent with the results of the semiautomatic CBMN assay, IR-induced chromosomal aberrations increased in a manner dependent on the copy number of *ATM* mutations (Fig. 3a and c). The variation of chromosomal aberrations in the genome-edited clones with each *ATM* genotype was more limited than those of cells derived from the A-T-affected family (Fig. 3b and d). Taken together, these results suggest that genome editing technology in human cultured cells might be a useful approach to evaluate genetic factors underlying individual differences in radiosensitivity within human populations.

## Discussion

Individual differences in radiosensitivity exist within human populations^2, 3^. For example, using the CBMN assay in peripheral lymphocytes, Scott *et al*. demonstrated that a certain proportion of healthy individuals exhibited mild radiosensitivity^5^. Previously, using the CBMN assay, we also demonstrated that B-cell lines from heterozygous carriers of a hyper-radiosensitive autosomal recessive disorder, Nijmegen breakage syndrome (NBS), were more radiosensitive than those from normal individuals^3^. Thus, we assumed that heterozygous mutations of hyper-radiosensitive recessive genetic disorders such as NBS and A-T are among the genetic factors underlying individual differences in radiosensitivity. To verify this, we here focused on *ATM* heterozygous mutations as a genetic factor of this kind, since a series of previous studies using A-T heterozygous carrier cells revealed that *ATM* heterozygous mutations have the capacity to increase radiosensitivity^12, 15^. Consistent with a previously reported study quantifying the changes in the number of foci of a DSB marker, γ-H2AX, in fibroblasts from the same A-T-affected family as used in this study^12^, we found that fibroblasts from the A-T heterozygous carriers showed significantly more IR-induced micronucleus formation and unstable chromosomal abnormalities including multicentric and ring chromosomes than those from normal individuals (Figs 1 and 3a,b, and Fig. S5). Importantly, variation in IR-induced micronucleus formation was detected among the A-T heterozygous carriers, even within the same family, suggesting that different genetic backgrounds within a family might affect the level of radiosensitivity of cells. Thus, it is technically difficult to clarify how much different genetic variants actually contribute to the individual differences in radiosensitivity, since the biological effects of the variants are interfered with by confounding factors such as gender, age, diverse genetic backgrounds and lifestyle factors including smoking and drinking alcohol. Unlike human primary cells from A-T-affected families, *ATM* heterozygous and homozygous mice with a uniform genetic background showed stable values of radiosensitivity as determined by a colony-survival assay^15^. It is therefore necessary to develop a highly sensitive system for detecting IR-induced DNA lesions in human cells with a candidate genetic variation in a uniform human genetic background.

In this study, we established an experimental flow combined with a semiautomatic CBMN assay and genome editing technology in a human cultured cell line as a unique system for evaluating genetic factors underlying individual differences in radiosensitivity. Genome editing technology has mainly been applied to human cell lines derived from cancer tissues, such as HEK293T cells and HCT116 cells, since they have intrinsic homologous recombination activity that is sufficient for the efficient isolation of genome-edited cells. Since it was reported that IR-induced cellular responses in human cells from normal tissues were quite different from those in cells from cancer tissues^19^, a normal human cell line should be more appropriate for an experimental model evaluating cellular radiosensitivity. We previously generated a gene knock-out hTERT-RPE1 cell line from normal retinal tissue using an artificial nuclease, TALEN, and a drug-resistant gene cassette contained in a targeting vector, but the efficacy for isolation of the targeted clones was low at approximately 1% of drug-resistant clones^20^. We thus attempted to design a procedure for site-specific gene insertion utilizing NHEJ activity, which is the dominant pathway of DNA repair in human cell lines. Maresca *et al*. inserted the artificial nuclease ZFN site located in the genome into a drug-resistant gene cassette plasmid vector, and cointroduced the ZFN and the targeting vector into human cultured cells to isolate the targeted clones with high efficacy^14^. They named the method ObLiGaRe, based on the Latin verb *obligate* (“to join to”). The ObLiGaRe approach has been applied in many species and cultured cells for gene knock-out and knock-in experiments^21, 22^. In this study, we combined the CRISPR/Cas9 system with the ObLiGaRe method to generate *ATM* ^−/−^ and *ATM* ^*+/−*^ hTERT-RPE1 cell clones. In the CRISPR-ObLiGaRe method in hTERT-RPE1 cells, biallelic targeting vector-inserted clones corresponding to *ATM* ^−/−^ cells were rare at 4.3% among the drug-resistant clones, while the monoallelic inserted clones were dominant at 71.6% (Table 2). Since 95.4% of monoallelic inserted clones carried the exact NHEJ-mediated insertions or deletions at the targeted *ATM* locus in a non- inserted allele, 72.5% of the drug-resistant clones were indeed *ATM* ^−/−^ cells. On the other hand, *ATM* ^*+/−*^ cells were generated at a rate of 3.3% of the drug-resistant clones (Table 2). Further investigations on the control of NHEJ activity are thus needed to isolate heterozygous clones efficiently using the CRISPR-ObLiGaRe method. However, the CRISPR-ObLiGaRe method is clearly a highly efficient gene-targeting method in the hTERT-RPE1 cell line.

Since the variations of IR-induced micronucleus formation and chromosome aberrations among *ATM* ^*+/−*^ hTERT-RPE1 cell clones were small compared with those in primary fibroblasts from A-T heterozygous carriers, the effect of *ATM* heterozygous mutations on radiosensitivity could be quantified using the formula α + β, which was shown to effectively monitor IR-induced micronucleus formation upon γ-ray irradiation at an intensity level of 1 Gy (Fig. 2e). Based on the data of the semiautomatic CBMN assay in *ATM*-edited cell clones, we concluded that *A-T* heterozygous null mutations had an effect of increasing cellular radiosensitivity by 2.6-fold, and suggested that they were indeed a genetic factor underlying individual differences in radiosensitivity within human populations. A number of genetic disorders associated with severe radiosensitivity have been reported, such as Lig-IV syndrome (*DNA ligase IV* mutations), A-T-like disorder (ATLD; *MRE11* mutations), NBS-like disorder (NBSLD; *Rad50* mutations), severe combined immunodeficiency (SCID; *DNA-PKcs* mutations) and Fanconi anaemia (*FANC* gene mutations). Heterozygous *BRCA1* and *BRCA2* mutations underlying hereditary breast and ovarian cancers (HBOCs) have also been reported to be involved in cellular radiosensitivity and cancer susceptibility. These genetic disorders are generally rare, while both heterozygous carriers of these mutations and HBOC patients are known to exist at rates of around 0.1–1%^23^. However, it was estimated that mutations conferring radiosensitivity might be present in a significant proportion of the whole population, such as at a rate of 5–15%^23, 24^, implying that these mutations might make a major contribution to the heterogeneity in radiosensitivity within human populations. The CRISPR-ObLiGaRe method is thus a useful tool for quantifying the effect of these mutations on radiosensitivity.

A number of epidemiological studies have shown that many single-nucleotide variants (SNVs) in DNA repair genes and their intergenic regions are associated with various cancer risks^25^, and that SNVs in inflammation-related genes (*TGF-β1*, *TNF*, and *ILs*), stress response-related genes (*MTHFR* and *HSPB1*) and angiogenesis-related genes (*VEGF*) are also involved in the toxicities of radiation therapy^26, 27^, suggesting that it will be difficult for researchers in the field of radiation science to determine whether or not they are involved in individual differences in radiosensitivity. Previously, we developed a TALEN-mediated two-step single-base-pair editing technology in human cultured cells. However, it was labour-intensive because it required multiple sets of TALENs and two rounds of drug selection^28^. A recent major approach of the SNV-knock-in technique involving the cointroduction of single-strand oligodeoxynucleotides (ssODN) with a candidate SNV and the CRISPR/Cas9 system into human cultured cells is a robust solution for quantifying the effects of SNVs on individual differences in radiosensitivity; however, it is not perfect because it is dependent on homologous recombination^29^. Notably, microhomology-mediated end joining (MMEJ)-assisted gene knock-in and NHEJ-mediated site-specific gene insertion, named PITCh (Precise Integration into Target Chromosome) and HITI (Homology-Independent Targeted Integration), respectively, were reported as homologous recombination-independent precise knock-in methods^30, 31^. If these methodologies and our previously reported two-step single-base-pair editing technology are merged, a unique approach can be established for evaluating the effect of candidate SNVs in human cultured cells from normal tissues. High throughput validation of the unique SNVs conserved among radiosensitive individuals by reverse genetics might provide correct diagnosis and convincing genetic markers to generate a personal radiation protection system for practical and clinical situations including radiation disasters, radiation therapy and CT imaging. Taking these approaches together, further improvement of genome-editing technology in human cultured cell lines with a uniform genetic background might enable further exploration of the genetic mechanisms underlying individual differences in radiosensitivity within human populations.

## Materials and Methods

### Cell cultures

Human primary fibroblasts (Coriell Institute, catalogue ID #516) were cultured in Dulbecco’s Modified Eagle Medium (DMEM, Gibco, Life Technologies) supplemented with 15% foetal bovine serum (FBS) and 50 mg/ml gentamycin. hTERT-RPE1 cells (ATCC CRL-4000) were maintained in DMEM with 10% FBS and 50 mg/ml gentamycin. All cells were grown at 37 °C in humidified air with 5% CO_2_.

### Plasmids

For the construction of an expression vector of both sgRNA targeting *ATM* gene exon 11 and spCas9, a pair of annealed oligodeoxynucleotides designed on the target site (5′-CTGACCACCAGTATAGTTCC-3′) with the overhangs of the BbsI restriction enzyme site were inserted into the pX330-U6-Chimeric_BB-CBh-hSpCas9 plasmid (Addgene, #42230). The targeting plasmid vector consisted of a BbsI restriction enzyme site flanked with a CMV promoter-driven *hsvTK-2A-Ne*o cassette as described previously^20^ in the pBluescript SK II^+^ backbone. A pair of oligodeoxynucleotides (5′-CTGACCACCAGTATAGTTCCAGG-3′) recognised by the CRISPR/Cas9 system for the targeting of *ATM* gene exon 11 were ligated into the targeting vector backbone mediated by the BbsI restriction enzyme site. Successful integration of oligodeoxynucleotides into each plasmid vector was verified by Sanger sequencing.

### Generation of *ATM*-edited hTERT-RPE1 cells using the CRISPR/ObLiGaRe method

A total of 2 × 10^5^ hTERT-RPE1 cells were seeded into one well of a six-well plate at 24 h before lipofection. Then, 10 ng of the targeting vector and 500 ng of the pX330 plasmid vector for the *ATM* gene exon 11 editing were transfected into the cells using Lipofectamine LTX (Thermo Fisher), in accordance with the manufacturer’s protocol. At 48 h after the transfection, the transfected cells were reseeded into 15-cm dishes and then subjected to selection using 2 mg/ml G418 (Nacalai Tesque). The drug-resistant cell colonies were then picked up on days 16–20 after transfection. These colonies were divided into two aliquots: one was transferred into a well of a 96-well plate for clonal expansion, while the other was lysed and used for PCR and direct-sequence genotyping.

### PCR-based and Sanger sequencing genotyping of the neomycin-resistant clones

PCR genotyping to screen for the *ATM*-edited hTERT-RPE1 cell clones was performed using extracted genomic DNA as a template and KOD-FX Neo DNA polymerase (TOYOBO) with three types of primer pair: the first primer pair consisted of *ATM* gene exon 11 forward *Fp* (5′-TCCTGCAGTATGCTGTTTGACTTTGGC-3′) and *ATM* gene exon 11 reverse *Rp* (5′-CTGTGAAGAATTGGAGGCACTTCTGTGC-3′) primers for detecting *ATM* gene exon 11; the second primer pair consisted of *ATM* gene exon 11 forward *Fp* and *Neo* ^*r*^-reverse *Np* (5′-GCGGATCTGACGGTTCACTAAACCAGC-3′) primers for detecting the forward insertion of the drug-resistant gene cassette into *ATM* gene; and the third primer pair consisted of *ATM* gene exon 11 reverse *Rp* and *Neo* ^*r*^-reverse *Np* primers for detecting the reversed insertion. PCR products were run on a 2.0% agarose gel. The wild-type-sized PCR products amplified with the third primer pair were directly sequenced using 3130 Genetic Analyzer (Applied Biosystems).

### Western blotting analysis

The asynchronized cells were lysed in RIPA buffer [50 mM Tris-HCl, pH 8.0, 150 mM NaCl, 1% NP-40 (v/v), 0.1% sodium dodecyl sulphate, 0.5% sodium deoxycholate, 0.5 mM PMSF, 2 ng/ml pepstatin A, 10 mg/ml leupeptin, 5 mg/ml aprotinin]. The lysates were sheared with a 21-gauge needle, incubated on ice for 20 min and subjected to centrifugation at 20,817 g for 15 min and 4 °C. Protein levels of ATM and GAPDH were quantified using an automated capillary-based western blotting system, namely, the device WES from Proteinsimple. All steps were performed with the manufacturer’s reagents in accordance with the user manual. Briefly, 4 μl of cell lysate was mixed with 1 μl of 5x fluorescent master mix containing dithiothreitol and heated at 95 °C for 5 min. The prepared cell lysates, primary and secondary antibodies, a biotinylated ladder and HRP chemiluminescent substrate were dispensed into designated wells in a 384-well assay plate. Separation, stacking and immobilization were automatically performed using a separation matrix for high-molecular-weight proteins (Standard Pack 3, 66–440 kDa; ProteinSimple) and for low-molecular-weight ones (Standard Pack 1, 12–230 kDa; ProteinSimple). The data were analysed with compatible Compass software in accordance with the ProteinSimple protocols. The primary antibodies used were anti-ATM rabbit monoclonal antibody (Abcam, ab32420) and anti-GAPDH mouse monoclonal antibody (Santa Cruz Biotechnology, sc-32233).

### Gamma irradiation

Cells cultured in six-well plates were irradiated with a γ-ray dose that ranged from 0.5 to 2 Gy (^137^Cs γ-ray source, 148 TBq, Gammacell 40 Exactor; Best Theractronics). The dose rate used was around 1 Gy/min.

### Semiautomatic cytochalasin-block micronucleus (CBMN) assay

A total of 2 × 10^4^ primary fibroblasts and 4 × 10^4^ hTERT-RPE1 cells were seeded on coverslips in one well containing 2 ml of growth medium in a six-well plate, incubated for 4 h at 37 °C to allow cell attachment, and then irradiated. After 48 h of incubation following γ-ray irradiation, 3 μg/ml cytochalasin B (Wako) was added and the mixture was incubated for 20 h at 37 °C. Cells were then fixed with 100% methanol for 20 min at −20 °C, briefly washed with PBS three times, and then incubated for 40 min at room temperature with blocking solution (1% bovine serum albumin; Sigma Aldrich). Next, the cells were stained with 4ʹ-6-diamidino-2-phenylindole (DAPI; 1:500 in 1% BSA) for 30 min at room temperature. These stained cells were scanned at 10× magnification with a Metafer 4 Scanning System comprising a Carl Zeiss Axioplan Imager Z1. The metafer classifier was activated on the Metafer MSearch platform. Captured images were analysed with the Metafer 4_MN program (MetaSystems). Criteria for selecting BN cells and MN were as previously described^4^. All scanned images of BN cells and MN were re-evaluated visually to exclude false positive and/or negative images of BN cells and MN.

The frequency of MN in each sample was assessed as the percentage of MN per total BN cells. Dose-response curves were analysed using a linear-quadratic equation (Y = c + αD + βD^2^), where Y is the yield of micronuclei, D is the radiation dose, α is a linear coefficient, β is a quadratic coefficient and c is the background frequency of MN. To quantify radiosensitivity, the linear-quadratic equation was transformed as described previously^17^ to the final form [(micronuclei frequency −c) = α + β] at a dose of 1 Gy: [α + β] reflects indeed cellular radiosensitivity. The extraction of each coefficient score was performed using Chromosomal Aberration Calculation Software (CABAS, freely available at http://www.pu.kielce.pl/ibiol/cabas)^16^.

### Chromosomal aberration analysis using PNA-FISH probes

Cells irradiated with γ-rays at 1 and 2 Gy were cultured for 24 h at 37 °C and arrested in metaphase with 0.1 mg/ml colcemid (Gibco) for 3 h at 37 °C. They were then treated with a hypotonic solution (0.075 M KCl) for 20 min at room temperature, and subsequently fixed with Carnoy solution (methanol:acetic acid, 3:1). Metaphase slides were prepared using Hanabi Metaphase Spreader. Hybridization with Cy3-labelled centromere probes (Panagene) and FITC-labelled telomere probes (Panagene) and counterstaining with DAPI in Vectashield mounting medium (Vector Laboratories) were performed as described previously. PNA-FISH-stained chromosome images were scanned at 10× and 63× magnification with the Metafer 4 Scanning System comprising a Carl Zeiss Axioplan Imager Z1. The PNA-FISH classifier was run in MSearch fluorescent mode. Captured images were analysed with Metafer 4_MN and Isis software (MetaSystems). Unrepaired DNA double-strand breaks in each sample were estimated from the data in PNA-FISH analysis using a previously described equation^32^.

### Statistical analysis

The experiments were performed independently three times, and the data are shown as mean ± s.e. Differences between groups were evaluated for statistical significance using Student’s *t*-test. Values of p < 0.05 were considered to be statistically significant.

## Electronic supplementary material

Supplementary Information
