# Supplementary material for: Evaluation of ATM heterozygous mutations underlying individual differences in radiosensitivity using genome editing in human cultured cells
Source: Sci Rep. 2017 Jul 20;7:5996. doi: 10.1038/s41598-017-06393-8 (PMC5519549; doi:10.1038/s41598-017-06393-8)
Supplement: Supplementary file 1 — Supplementary Information [file 41598_2017_6393_MOESM1_ESM.pdf]

Supplementary Information

Figures S1 to S7 and Tables S1 and S2

Evaluation of *ATM* heterozygous mutations underlying individual differences in radiosensitivity using genome editing in human cultured cells

Ekaterina Royba[1], Tatsuo Miyamoto[1], Silvia Natsuko Akutsu[1], Kosuke Hosoba[1], Hiroshi Tauchi[2], Yoshiki Kudo[3], Satoshi Tashiro[4], Takashi Yamamoto[5] and Shinya Matsuura[1*]

[1]*Department of Genetics and Cell Biology, Research Institute for Radiation Biology and Medicine, Hiroshima University, Hiroshima 734-8553, Japan*

[2]*Department of Biological Sciences, Faculty of Science, Ibaraki University, Mito 310-8512, Japan*

[3]*Department of Obstetrics and Gynecology, Graduate School of Biomedical Sciences, Hiroshima University, Hiroshima 734-8551, Japan*

[4]*Department of Cellular Biology, Research Institute for Radiation Biology and Medicine, Hiroshima University, Hiroshima 734-8553, Japan*

[5]*Department of Mathematical and Life Sciences, Graduate School of Science, Hiroshima University, Higashi-Hiroshima 739-8526, Japan*

Correspondence: e-mail: shinya@hiroshima-u.ac.jp

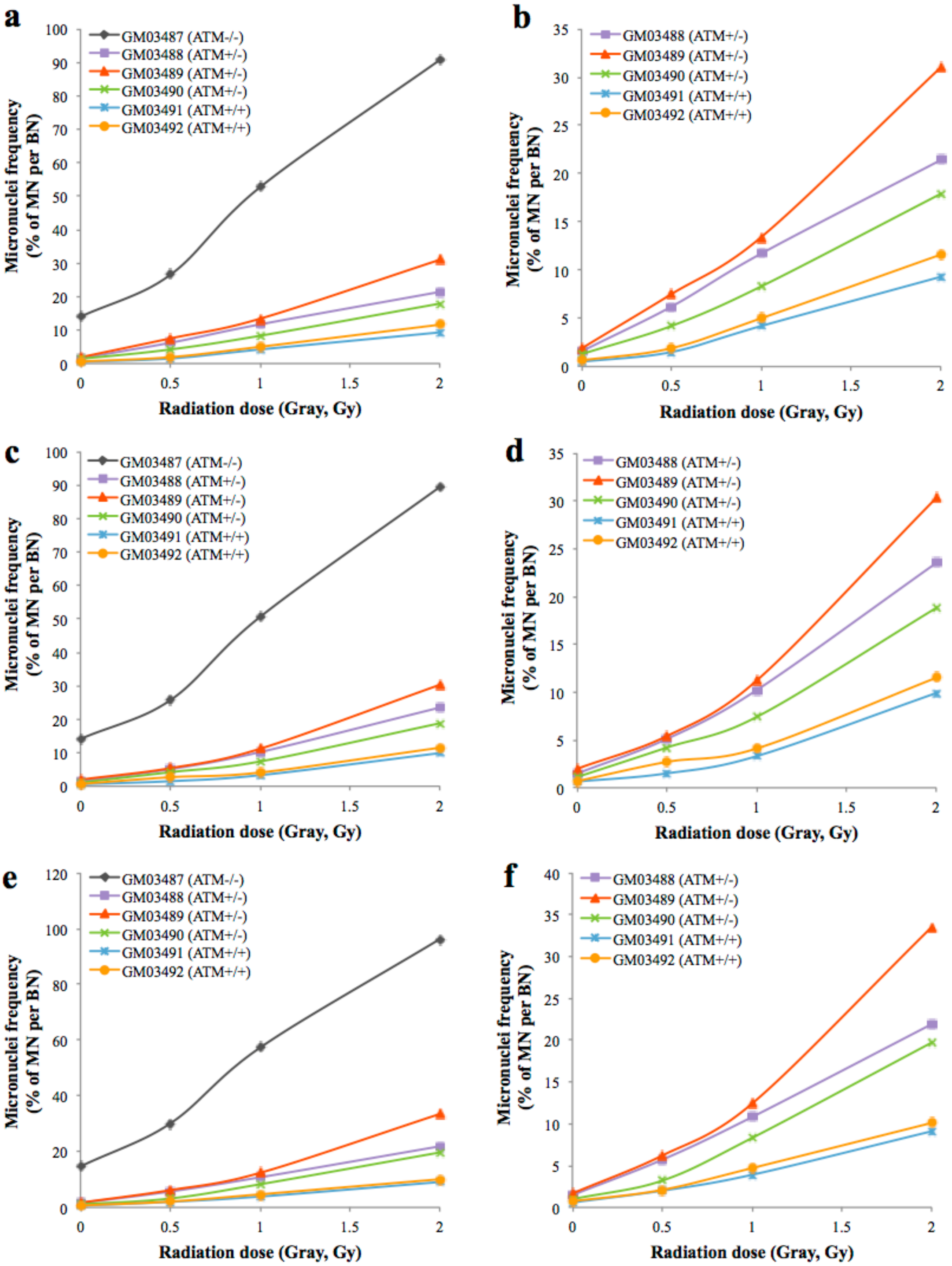

Figure S1. Three independent CBMN assays in primary fibroblasts from an A-T-affected family. (a), (c) and (e) Three graphs showing the results of three independent CBMN assays. Percentage of IR-induced MN formation in fibroblasts from all members of the A-T-affected family (>1000 BN cells, >50 BN cells only in A-T patient fibroblasts). **(b) (d) and (f)** The graphs from (a), (c) and (e), respectively, with magnification of the Y-axis including the percentage of IR-induced MN formation in fibroblasts derived from A-T heterozygous carriers and unaffected individuals.

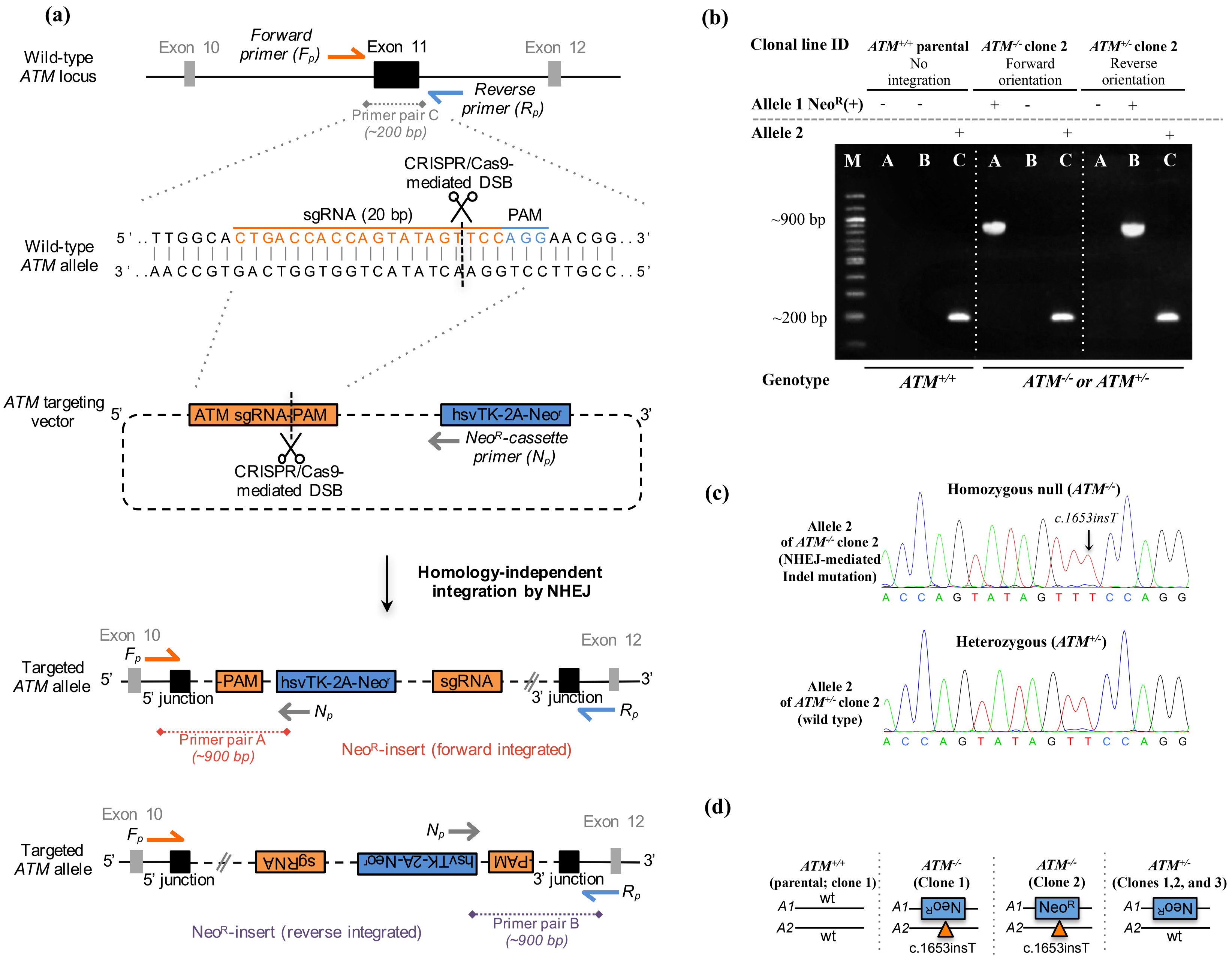
(a)
Wild-type ATM locus
Exon 10
Forward primer (F_p)
Exon 11
Exon 12
Reverse primer (R_p)
Primer pair C (~200 bp)
CRISPR/Cas9-mediated DSB
sgRNA (20 bp)
PAM
Wild-type ATM allele
5'..TTGGCACTGACCACCAGTATAGTTCCAGGAACGG..3'
3'..AACCGTGACTGGTGGTCATATCAAGGTCCTTGCC..5'
ATM targeting vector
ATM sgRNA-PAM
hsvTK-2A-Neo^r
Neo^R-cassette primer (N_p)
CRISPR/Cas9-mediated DSB
Homology-independent integration by NHEJ
Targeted ATM allele
F_p
5' junction
PAM
hsvTK-2A-Neo^r
sgRNA
3' junction
N_p
R_p
Primer pair A (~900 bp)
Neo^R-insert (forward integrated)
Neo^R-insert (reverse integrated)
Primer pair B (~900 bp)
(b)
Clonal line ID
ATM+/+ parental No integration
ATM-/- clone 2 Forward orientation
ATM+/- clone 2 Reverse orientation
Allele 1 Neo^R(+) - - + - - +
Allele 2 + + +
M A B C A B C A B C
~900 bp
~200 bp
Genotype
ATM+/+
ATM-/- or ATM+/-
(c)
Homozygous null (ATM-/-)
c.1653insT
Allele 2 of ATM-/- clone 2 (NHEJ-mediated Indel mutation)
A C C A G T A T A G T T T C C A G G
Heterozygous (ATM+/-)
Allele 2 of ATM+/- clone 2 (wild type)
A C C A G T A T A G T T C C A G G
(d)
ATM+/+ (parental; clone 1)
ATM-/- (Clone 1)
ATM-/- (Clone 2)
ATM+/- (Clones 1,2, and 3)
A1
A2
wt
Neo^R
c.1653insT

Figure S2. CRISPR-ObLiGaRe method generated ATM-edited hTERT-RPE1 cell lines with a uniform genetic background.

(a) The CRISPR/ObLiGaRe-mediated genome-editing strategy. *ATM* gene exon 11 and the targeting vector containing an *hsvTK-2A-Neo*^{r} selection cassette were cleaved in hTERT-RPE1 cells using a set of Cas9-nuclease and the sgRNA targeting for *ATM* gene exon 11. The linearized targeting vector was integrated into *ATM* gene exon 11 in an NHEJ-dependent manner. The orientation of the targeting vector upon insertion could not be controlled. For genotyping of the targeted site in positive drug-selected clones, PCR genotyping and direct sequencing were combined. **(b)** PCR genotyping step: the integration of a drug-resistant cassette was evaluated by PCR using the primer pairs A and B as shown in (a). *ATM* exon 11 without a gene cassette was amplified by PCR using primer pair C denoted in (a). An agarose-electrophoresis image showed the presence or absence of the insertion of a targeting vector into *ATM* gene exon 11 in the parental RPE1 cell, *ATM*$^{-/-}$ clone 2 and *ATM*$^{+/-}$ clone 2. **(c)** Direct sequencing step: Insertions/deletions in an allele without a drug-resistant cassette were directly checked by Sanger sequencing. *ATM*$^{-/-}$ clone 2 contained a 1-bp insertion (c.1653 insT, p.V551X) at a CRISPR/Cas9-mediated DSB site. **(d)** Schematic representation of the final genotype in *ATM*-edited hTERT-RPE1 cell clones generated by the CRISPR/ObLiGaRe method. For details, see Table 1.

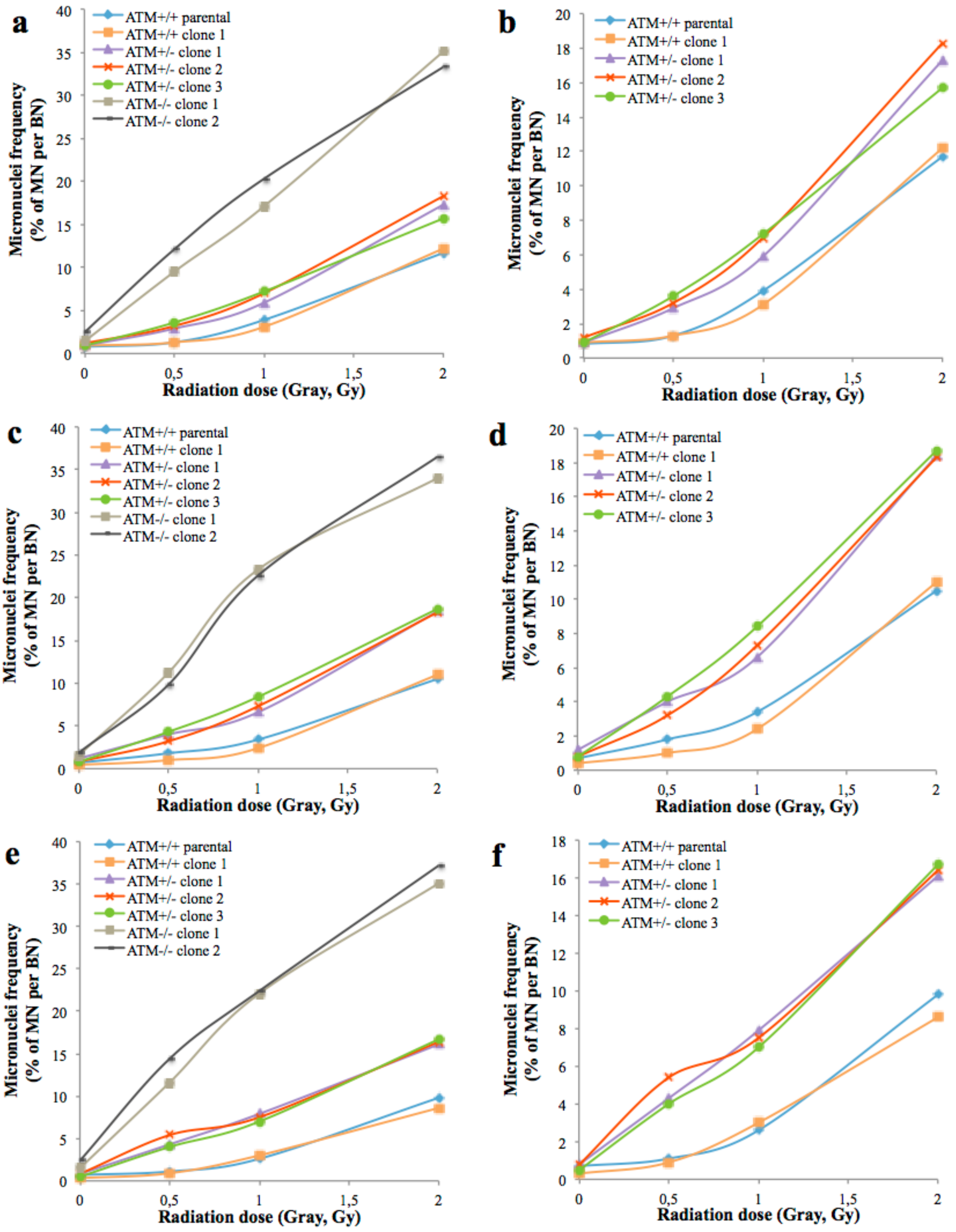

Figure S3. Three independent CBMN assays in *ATM*-edited hTERT-RPE1 cell clones.

(a), (c) and (e) Three graphs showing the results of three independent CBMN assays. Percentage of IR-induced MN formation in *ATM*-edited cell clones (>1000 BN cells, >50 BN cells only in $ATM^{-/-}$ cell clones). **(b), (d) and (f)** The graphs from (a), (c) and (e), respectively, with magnification of the Y-axis including the percentage of IR-induced MN formation in $ATM^{+/-}$ and $ATM^{+/+}$ cell clones.

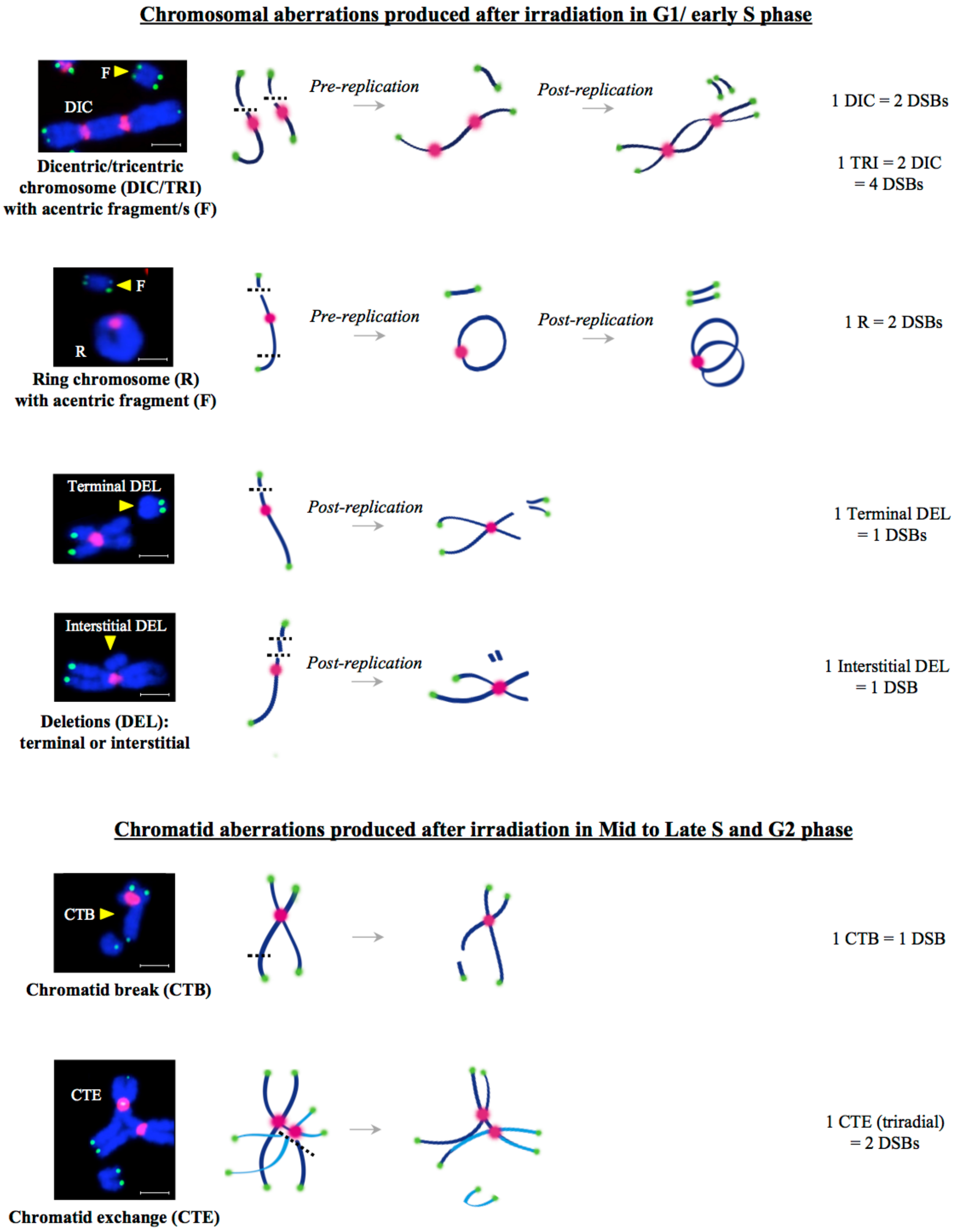

Figure S4. Evaluation of unrepaired DNA DSBs from chromosomal aberrations detected by PNA-FISH analysis. Since the pattern of chromosomal aberrations is dependent on the number of unrepaired DSBs, we considered the process of formation of each such aberration to quantify unrepaired DSBs. Scale bars: 2 μm.

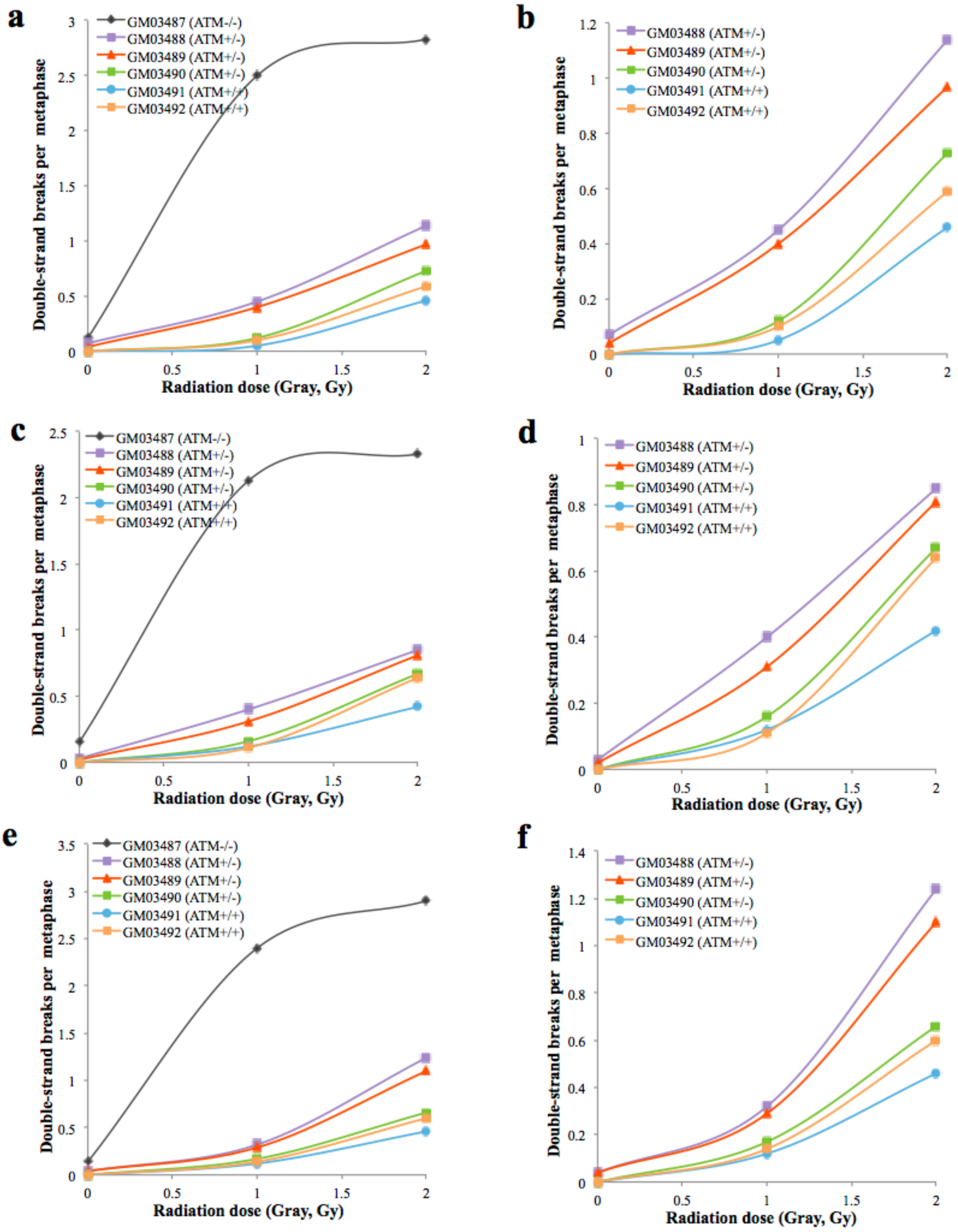

Figure S5. Centromere/telomere PNA-FISH analysis in the primary fibroblasts from the A-T-affected family. (a), (c) and (e) Raw data for three independent trials in the primary fibroblasts. **(b), (d) and (f)** from (a), (c) and (e), respectively, with magnification of the Y-axis for three independent trials in the fibroblasts from A-T heterozygous carrier and normal individuals.

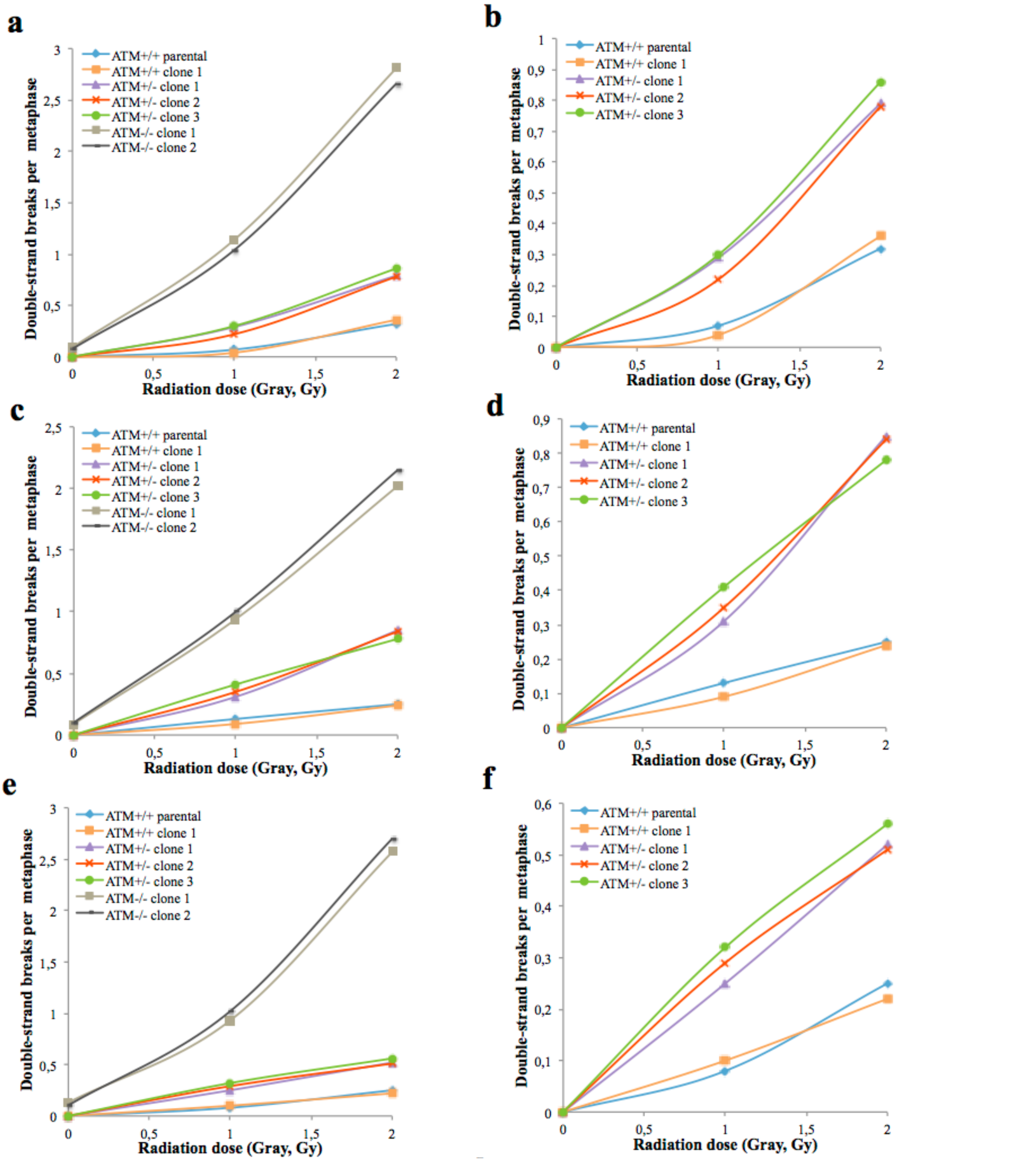

Figure S6. Centromere/telomere PNA-FISH data in *ATM*-edited hTERT-RPE1 cell lines. (a), (c) and (e) Raw data for three independent trials in *ATM*-edited hTERT-RPE1 cell lines. **(b), (d)** and **(f)** Raw data from (a), (c) and (e), respectively, with magnification of the Y-axis for three independent trials in $ATM^{+/-}$ and $ATM^{+/+}$ cell clones.

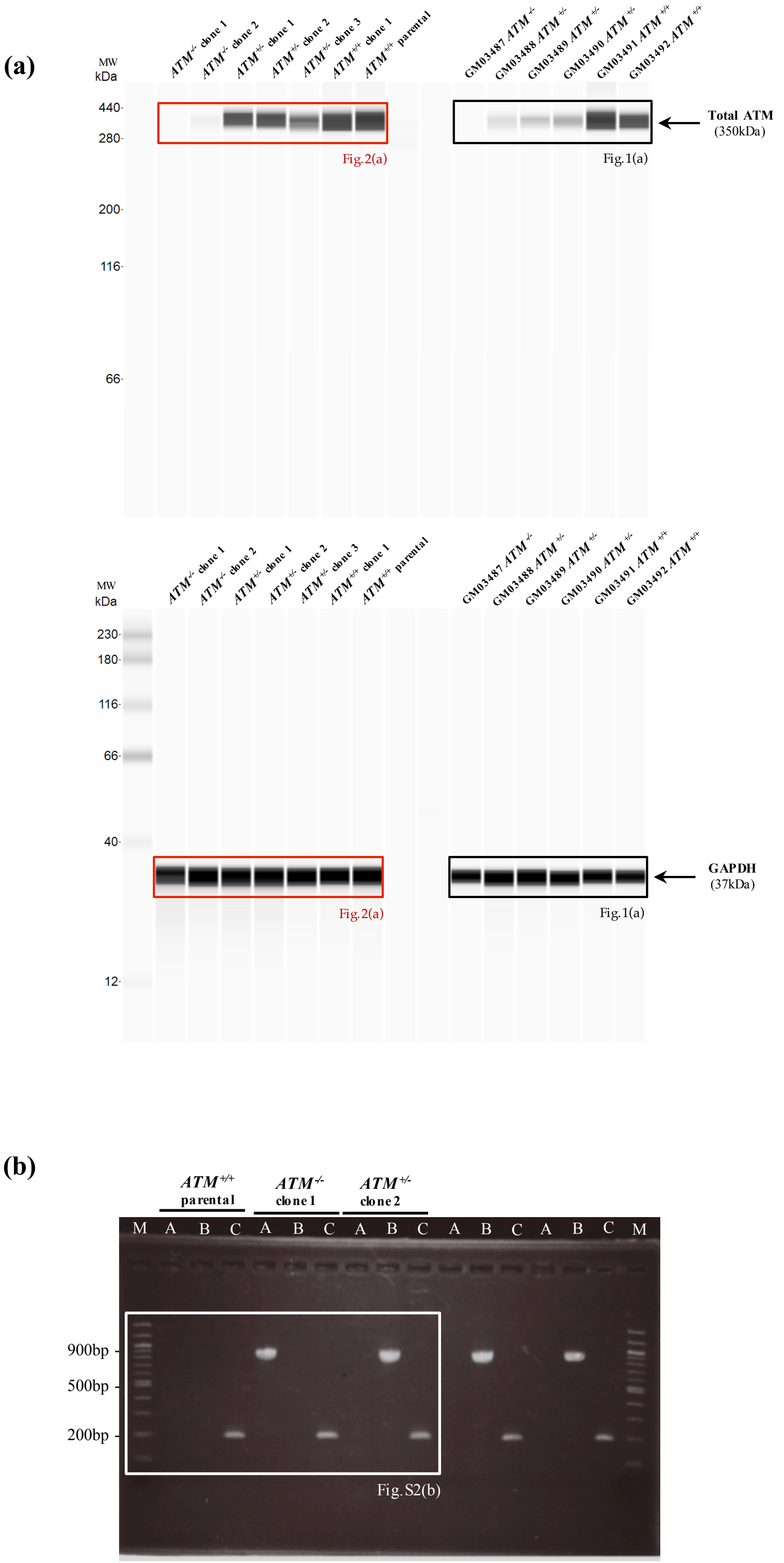

Figure S7. Full-scan of western blots and agarose gel electrophoresis. (a) Raw data of Fig. 1a and Fig. 2a captured using an automated capillary-based western blotting system. **(b)** Raw image of agarose gel electrophoresis in Fig. S2b.

Fibroblast cell line ID (Coriell Institute)	Donor	*ATM* gene mutation (Transcript # ATM-201, ENST00000278616.8)			Genotype
		ATM exon #	DNA sequence change	Amino acid change	
GM03487	Male, 8 y.o., proband	Exon 9<br>Exon 56	c.1141ins4<br>c.8266A>T	[S381X]<br>[K2756X]	$ATM^{-/-}$
GM03488	Male, 41 y.o., father	Exon 9	c.1141ins4	[S381X]	$ATM^{+/-}$
GM03489	Female, 37 y.o., mother	Exon 56	c.8266A>T	[K2756X]	$ATM^{+/-}$
GM03490	Female, 16 y.o., sister	Exon 9	c.1141ins4	[S381X]	$ATM^{+/-}$
GM03491	Female, 15 y.o., sister	-	Wild type	Wild type	$ATM^{+/+}$
GM03492	Male, 7 y.o., brother	-	Wild type	Wild type	$ATM^{+/+}$

Table S1. Genetic information about *ATM* gene in six members of the A-T-affected family examined in this study.

Cell line ID	*ATM* exon #	Allele 1 *(Obligare-mediated Neo^R cassette integration)*	Allele 2 *(CRISPR/Cas9-mediated indel mutations)* (Transcript # ATM-201, ENST00000278616.8)		Genotype
			DNA sequence change	Amino acid change	
ATM$^{-/-}$ clone 1	Exon 11	Neo+ (reverse orientation)	c.1653insT	[V551X]	*ATM*$^{-/-}$
ATM$^{-/-}$ clone 2	Exon 11	Neo+ (forward orientation)	c.1653insT	[V551X]	*ATM*$^{-/-}$
ATM$^{+/-}$ clone 1	Exon 11	Neo+ (reverse orientation)	Wild type	Wild type	*ATM*$^{+/-}$
ATM$^{+/-}$ clone 2	Exon 11	Neo+ (reverse orientation)	Wild type	Wild type	*ATM*$^{+/-}$
ATM$^{+/-}$ clone 3	Exon 11	Neo+ (reverse orientation)	Wild type	Wild type	*ATM*$^{+/-}$
ATM$^{+/+}$ clone 1	-	Neo- (wild type) plus Neo+ (random integration)	Wild type	Wild type	*ATM*$^{+/+}$
ATM$^{+/+}$ parental	-	Neo- (wild type)	Wild type	Wild type	*ATM*$^{+/+}$

Table S2. Genetic information of *ATM*-edited hTERT-RPE1 cell clones.
